# Prediction of new-onset migraine using clinical-genotypic data from the HUNT Study: a machine learning analysis

**DOI:** 10.1186/s10194-025-02014-2

**Published:** 2025-04-07

**Authors:** Fahim Faisal, Antonios Danelakis, Marte-Helene Bjørk, Bendik Winsvold, Manjit Matharu, Parashkev Nachev, Knut Hagen, International Headache Genetics Consortium, Erling Tronvik, Anker Stubberud

**Affiliations:** 1 Norhead Norwegian Centre for Headache Research, NTNU Norwegian University of Science and Technology, Trondheim, Norway; 2 Department of Neuromedicine and Movement Science, NTNU Norwegian University of Science and Technology, Trondheim, Norway; 3 Department of Computer Science, NTNU Norwegian University of Science and Technology, Trondheim, Norway; 4 Department of Clinical Medicine, University of Bergen, Bergen, Norway; 5 Department of Neurology, Haukeland University Hospital, Bergen, Norway; 6 Department of Research and Innovation, Division of Clinical Neuroscience, Oslo University Hospital, Oslo, Norway; 7 Department of Neurology, Oslo University Hospital, Oslo, Norway; 8 HUNT Center for Molecular and Clinical Epidemiology, Department of Public Health and Nursing, Faculty of Medicine and Health Sciences, NTNU Norwegian University of Science and Technology, Trondheim, Norway; 9 UCL Queen Square Institute of Neurology, University College London, London, UK; 10 Neuroclinic, St Olav University Hospital, Trondheim, Norway

## Abstract

**Background:**

Migraine is associated with a range of symptoms and comorbid disorders and has a strong genetic basis, but the currently identified risk loci only explain a small portion of the heritability, often termed the “missing heritability”. We aimed to investigate if machine learning could exploit the combination of genetic data and general clinical features to identify individuals at risk for new-onset migraine.

**Method:**

This study was a population-based cohort study of adults from the second and third Trøndelag Health Study (HUNT2 and HUNT3). Migraine was captured in a validated questionnaire and based on modified criteria of the International Classification of Headache Disorders (ICHD) and participants underwent genome-wide genotyping. The primary outcome was new-onset migraine defined as a change in disease status from headache-free in HUNT2 to migraine in HUNT3. The migraine risk variants identified in the largest GWAS meta-analysis of migraine were used to identify genetic input features for the models. The general clinical features included demographics, selected comorbidities, medication and stimulant use and non-headache symptoms as predictive factors. Several standard machine learning architectures were constructed, trained, optimized and scored with area under the receiver operating characteristics curve (AUC). The best model during training and validation was used on unseen test sets. Different methods for model explainability were employed.

**Results:**

A total of 12,995 individuals were included in the predictive modelling (491 new-onset cases). A total of 108 genetic variants and 67 general clinical variables were included in the models. The top performing decision-tree classifier achieved a test set AUC of 0.56 when using only genotypic data, 0.68 when using only clinical data and 0.72 when using both genetic and clinical data. Combining the genotype only and clinical data only models resulted in a lower predictivity with an AUC of 0.67. The most important clinical features were age, marital status and work situation as well as several genetic variants.

**Conclusion:**

The combination of genotype and routine demographic and non-headache clinical data correctly predict the new onset of migraine in approximately 2 out of 3 cases, supporting that there are important genotypic-phenotypic interactions partaking in the new-onset of migraine.

**Supplementary Information:**

The online version contains supplementary material available at 10.1186/s10194-025-02014-2.

## Background

A large proportion of individuals with migraine suffer for a long time without a proper diagnosis and adequate treatment [1–3]. At present, there are few tools that may accurately predict the new-onset of migraine in adult age [4]. Predicting individuals at risk of developing migraine and recognizing the early onset of the condition can enhance prevention, reduce misdiagnosis, and avoid costly, unnecessary investigations and ineffective treatments that fail to alleviate patient burden [5, 6]. Early and efficient treatment of migraine can also halt or limit progression and disability [7].

Risk factors for migraine are both genetic and environmental, but the total effect in one individual seems unpredictable. A strong heritability of around 50% is established [8], yet the hereto largest meta-analysis of genome-wide association studies (GWAS), identifying 123 risk loci for migraine [9], only explains 11.2% of the heritability. This suggests genetic risk may be conveyed in non-linear interactions between loci opaque to the simple statistical models traditionally used to analyze GWAS data and derive polygenic risk scoring systems. Among the environmental factors, stress is quoted as a risk factor for new-onset migraine [10, 11]. Moreover, we know that there are bidirectional associations between migraine and neurological, psychiatric, cardiovascular, gastrointestinal, endocrine and immunological disorders [12], all of which can be hypothesized to impact the risk of new-onset of migraine.

Because each of these factors alone are relatively weakly associated with migraine in absolute terms, it is probable that accounting for the complex relationship between many of them will provide the best predictive power for identifying individuals at risk. Unlike conventional statistical methods, machine learning can support models with sufficient expressivity to capture complex relations distributed across wide fields of putatively causal factors. The primary goal of this study was to investigate if it is possible to predict which individuals develop new-onset migraine using both sociodemographic, clinical and genetic information in machine learning models. Secondly, we aimed to elucidate which of these factors are most important for predicting new-onset migraine, and to quantify the relative contribution of genetic versus clinical domains.

## Method

### Study design

**Fig. 1:**
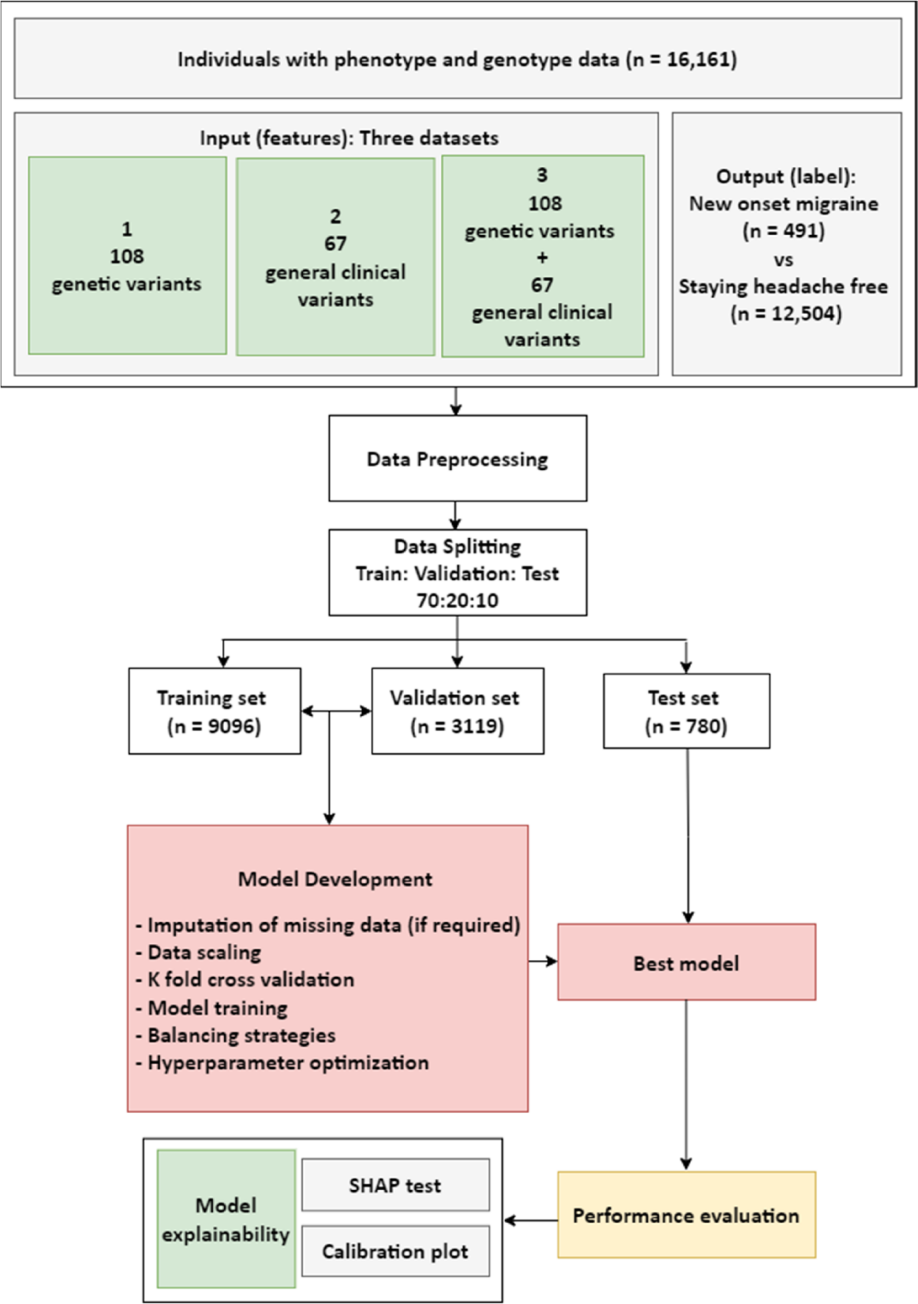
Schematic explanation of the general study design

This study was a population-based longitudinal cohort study of adults from the second and third Trøndelag Health Studies (HUNT2 and HUNT3). We explored and evaluated an extensive machine learning analysis utilizing genetic and clinical data from HUNT2. The overall study design is demonstrated in Fig. 1. Machine learning models were developed to predict the new-onset of migraine.

### Data sources and data management

#### The Trøndelag Health Study (HUNT)

The Trøndelag Health Study (HUNT) is a large health survey carried out in four waves between 1984 and 2017 until now [13]. Among them, HUNT2 was carried out in 1995–1997 [14] and HUNT3 in 2006–2008 [15]. All inhabitants in Nord-Trøndelag County were invited to participate. Clinical assessments, biological samples, and self-administered questionnaires were used to gather data. Participants gave thorough details on their sociodemographic characteristics, lifestyle, and medical history. Clinical personnel also performed physical exams and took blood samples which were then examined to find biomarkers and for genome-wide genotyping.

#### Genotype data

A total of 71,680 individuals were genotyped at the Genomics-Core Facility at the Norwegian University of Science and Technology using three different versions of the Illumina HumanCoreExome microarray [16]. The complete methods for imputation and quality control of genotype data are described in detail elsewhere [17]. We identified the available genotyped variant or imputed dosages in our data among the 123 risk loci identified in a recent large GWAS meta-analysis (base data/discovery data set) [9]. The imputed dosage refers to the estimated variant derived through imputation based on the surrounding known genotypes and haplotype, rather than direct observation of the variant. A total of 108 risk variants were available in our study population (target data).

#### Outcome definition

The outcome of interest was the development of new-onset migraine in individuals who were asymptomatic at the time of HUNT2 and subsequently reported migraine during HUNT3. Individuals that were headache-free in HUNT2, and then reported a migraine phenotype headache in HUNT3 were considered cases. Individuals that were headache-free in both HUNT2 and HUNT3 were considered controls. Phenotype assignment was made using validated modified ICHD criteria based on the HUNT questionnaires [18, 19]. Details of the phenotype assignment are reported elsewhere [18, 19]. Subjects with a diagnosis of migraine registered in the national hospital registry prior to the launch of HUNT2 were excluded as they would not represent a true new-onset of migraine.

#### Data preprocessing

We created three main datasets with differing data combinations to be inputted into the models: (1) only genotype data; (2) only general clinical variables collected in HUNT2 and (3) combined genotype and general clinical data (Fig. 1). The selection of general clinical variables to include in the predictive models was done in an incremental fashion. Among all available variables in HUNT2, those that had a known [20, 21] hypothesized or plausible relationship with migraine were evaluated. This included data on demographics, socio-economic status, history of familial diseases, work and education, cardiovascular, neurological, musculoskeletal and gynecological diseases, mental health, use of stimulants like alcohol and nicotine, use of selected medication, sleep patterns, and exercise amounting to 72 variables. Thereafter, variables with an unacceptable degree of missingness (> 20%) were omitted (n = 5). 67 general clinical variables were included in the predictive modelling. Table 1 gives a summary of variables. Supplementary Table A lists all the included variables.

**Table 1:** Subject Characteristics of controls and cases based on data from HUNT2

	Controls (*n* = 12,504)	Cases (*n* = 491)
Age, year, mean (SD)	50.18 (13.42)	38.94 (11.90)
Sex, male (%)	6560 (52.46)	182 (37.06)
Height, cm, mean (SD)	171.51 (9.15)	170.62 (8.35)
Weight, kg, mean (SD)	77.44 (13.25)	74.37 (13.30)
Waist Circumference, cm, mean (SD)	86.42 (10.92)	82.71 (11.41)
Hip Circumference, cm, mean (SD)	101.99 (7.28)	100.72 (8.05)
Body mass index, kg/m^2^, mean (SD)	26.27 (3.70)	25.49 (3.88)
Systolic Blood Pressure, mm Hg, mean (SD)	136.76 (19.46)	126.57 (15.17)
Diastolic Blood Pressure, mm Hg, mean (SD)	80.34 (11.39)	75.88 (9.47)
Blood Pressure Medication, with medication (%)	1194 (9.57)	20 (1.10)
Pulse, bpm, mean (SD)	70.83 (12.41)	72.25 (12.47)
Serum Cholesterol, mmol/L, mean (SD)	5.94 (1.22)	5.49 (1.17)
Serum HDL Cholesterol, mmol/L, mean (SD)	1.40 (0.39)	1.44 (0.40)
Serum Triglycerides, mmol/L, mean (SD)	1.73 (1.11)	1.52 (0.94)
Non-fast Serum Glucose, mmol/L, mean (SD)	5.40 (1.30)	5.07 (0.86)
Marital status, n (%)		
- Married	9002 (72.13)	268 (54.69)
- Unmarried	2128 (17.05)	179 (36.53)
- Divorced	584 (4.67)	28 (5.71)
- Widow/er	634 (5.08)	8 (1.63)
- Separated	132 (1.05)	7 (1.42)
Education, n (%)		
- High school, intermediate school, vocational school, 1–2 years high school	4350 (35.52)	201 (41.61)
- Primary school 7–10 years, continuation school, folk high school	4039 (32.97)	90 (18.63)
- University or other post-secondary education, less than 4 years	1685 (13.76)	79 (16.35)
- University/college, 4 years or more	1217 (9.77)	44 (9.11)
- University qualifying examination, junior college, A levels	957 (7.81)	69 (14.28)
Paid work, n (%)	9357 (75.25)	405 (83.33)
Family History of Cardiovascular Disease, n (%)	5739 (45.88)	173 (35.23)
Self-Reported Health, n (%)		
- Very good	2630 (21.18)	129 (26.38)
- Good	7731 (62.26)	279 (57.05)
- Not so good	1981 (15.95)	77 (15.74)
- Poor	76 (0.61)	4 (0.82)
No. of Gastrointestinal Symptoms Last Year, n (%)	0 = 6983 (55.84) 1 = 3040 (24.31) 2 = 1001 (8) > = 3 = 263 (2.1)	0 = 230 (46.84) 1 = 134 (27.29) 2 = 62 (12.62) > = 3 = 31 (6.31)
Estrogen and/or Progesterone, with birth control pills and/or systemic estrogen (%)	2972 (58.98)	236 (78.14)
Still Menstruation, n (%)	2897 (49.79)	248 (81.31)
Musculoskeletal Pain, n (%)	5055 (40.51)	191 (38.98)
Neck Pain, n (%)	2126 (20.16)	103 (23.41)
Anxiety and Depression, mean (SD)	6.46 (4.76)	6.85 (5.25)
Satisfied Life Situation, n (%)		
- Very Satisfied	1920 (15.50)	90 (18.44)
- Satisfied	4399 (35.52)	166 (34.02)
- Somewhat satisfied	4679 (37.78)	158 (32.37)
- Neither satisfied nor dissatisfied	1219 (9.84)	64 (13.11)
- Somewhat dissatisfied	116 (0.93)	6 (1.22)
- Dissatisfied	28 (0.22)	3 (0.61)
- Very dissatisfied	23 (0.18)	1 (0.2)
Smoking Status, n (%)		
- Never smoked daily	5842 (47.04)	232 (47.54)
- Ex-smoker daily	3752 (30.21)	144 (29.51)
- Current smoker daily	2824 (22.74)	112 (22.95)
Non-drinker of Alcohol, n (%)	1072 (8.65)	41 (8.41)
Light Exercise, n (%)		
- Less than 1 h - 1–2 h - 3 h or more	1811 (17.55) 4378 (42.43) 4127 (40.01)	77 (18.37) 171 (40.81) 171 (40.81)
Hard Exercise, n (%)		
- Less than 1 h	2584 (41.46)	110 (40)
- 1–2 h	2452 (39.34)	108 (39.27)
- 3 h or more	1196 (19.19)	57 (20.72)
Sleep Issues Last 1–3 Months, n (%)	4641 (37.48)	178 (36.33)
No. of Comorbidities, n (%)	0 = 8712 (69.67) 1 = 2033 (16.25) 2 = 469 (3.75) > = 3 = 146 (1.16)	0 = 360 (73.31) 1 = 81 (16.49) 2 = 17 (3.46) > = 3 = 6 (1.22)

*SD* standard deviation

Evaluation of the type of missingness was checked based on their common patterns and investigating associations to other variables, including the outcome variables, as (1) random, or (2) not at random. Data missing not at random was imputed based on clinical reasoning and assessment of the most probable reason for missingness. This included detailed manual and analytical review of the questionnaires, the subsequent data handling procedures and the data structure. The details for imputation strategies for each variable are provided in Supplementary Table B. A series of imputation techniques for the variables missing at random were implemented and evaluated by assessing their impact on the model's scoring metric. This included the simple mean and median imputers, and multiple imputation techniques including iterative imputers, k-nearest neighbors, multiple imputations by chained equations (MICE) [22], and DataWig [23].

The genetic variants included in the modelling were those available in our dataset (n = 108) among the 123 identified genetic risk variants [9]. Different methods for data representation of the genotype data were evaluated and included (1) using the crude genotyped or imputed dosage; (2) converting imputed dosages to the nearest integer (i.e. representing a genotype); and (3) converting the imputed dosages to the nearest integer and using the difference between the imputed dosage and the integer as a separate data point for uncertainty measure. One-hot-encoding representation of the genetic variants was also evaluated.

The dataset was split in a randomized manner into stratified training, validation, and test sets, with a 7: 2: 1 ratio for training set to validation set to test set. The stratification ensured even distribution of cases and controls in the training, validation, and test sets. Partitions were kept separate during training. Data was standardized by subtracting the mean and dividing by the standard deviation. No clipping was required as the distribution was well behaved. Class imbalance was addressed with multiple balancing strategies (see below).

### Predictive modelling

Several standard machine learning architectures were applied and analyzed, namely Logistic Regression, Support Vector classifier, Stochastic Gradient Descent, Naive Bayes, Decision Tree classifier, Random Forest, Gradient Boosting, Extreme Gradient Boosting, Ada Boost, Gaussian Process, Extra Tree classifier, Bagging classifier, Linear Discriminant Analysis, LightGBM classifier, k Nearest Neighbors classifier. Owing to the high degree of class imbalance, the cases were also evaluated as an anomalies for which anomaly detection algorithms such as epileptic envelope, isolation forest, one class support vector machine and local outlier were explored. Class imbalances were addressed using several different balancing strategies such as oversampling, under-sampling and Synthetic Minority Oversampling Technique (SMOTE) [24]. Models were trained on the train dataset and evaluated throughout the model development process by ten-fold cross validation on the training set. The validation set was used to evaluate the impact of changes to the modeling pipeline such as sampling strategies, data scaling and imputation. For the best candidate models, hyperparameters were optimized with a Randomized search with 100 iterations and Grid search strategy with promising parameter ranges identified in the randomized search. The impact of hyperparameter tuning was evaluated in cross-validation and the validation set.

The area under the receiver operating characteristics curve (AUC) was used as the primary scoring metric. We also calculated balanced accuracy, sensitivity and specificity. The top performing model was decided on the basis of a combination of cross-validated and validation set performance. This top performing model was applied to the held-out test set to quantify out of sample performance. We used mean and standard deviation (SD) for normal distributions and median with interquartile range (IQR) for the non-parametric distributions. Data were reported as means, standard deviations (SD), medians and interquartile ranges (IQR). Bootstrapping was used to derive 95% confidence intervals (CI) for the test set AUCs.

The modelling strategy was applied separately to the datasets including only genotype data, only general clinical data and combined genotype and general clinical data. The combined data was intended to take into account interactions between genetics and phenotype/environment. Finally, we evaluated voting classifiers that “added” the best predictive models using only genotype data and only clinical data, to compare additive versus interactive effects of genetics and phenotype/environment.

### Model explainability

For the best models, we calculated the Shapley values and represented them graphically by creating a summary plot based on SHAP. This method uses Shapley values to quantify the impact of every feature on the prediction by the model, providing interpretability for complex methods of machine learning. SHAP assigns each feature an importance score that shows how much it affected its predictions [25]. The SHAP summary plot demonstrates how each variable positively or negatively contributed to the model output and lends clarity to the strength and direction of predictions and the relative importance of one factor over the others across the dataset. A calibration plot with reliability diagram was also created of the combined model to visualize the model calibration and generalizability.

This is the primary machine learning analysis of new-onset migraine prediction with clinical and genotypic data from the HUNT Study. All statistical analyses were performed, and figures were made using Python v3.10 (Python Software Foundation) with the following open-source packages: matplotlib 3.6.1; numpy 1.23.4; pandas 1.5.0; scikit-learn 1.1.2; scikit-optimize 0.9.0; seaborn 0.12.0 and shap 0.41.0.

## Result

### Demographics

**Fig. 2:**
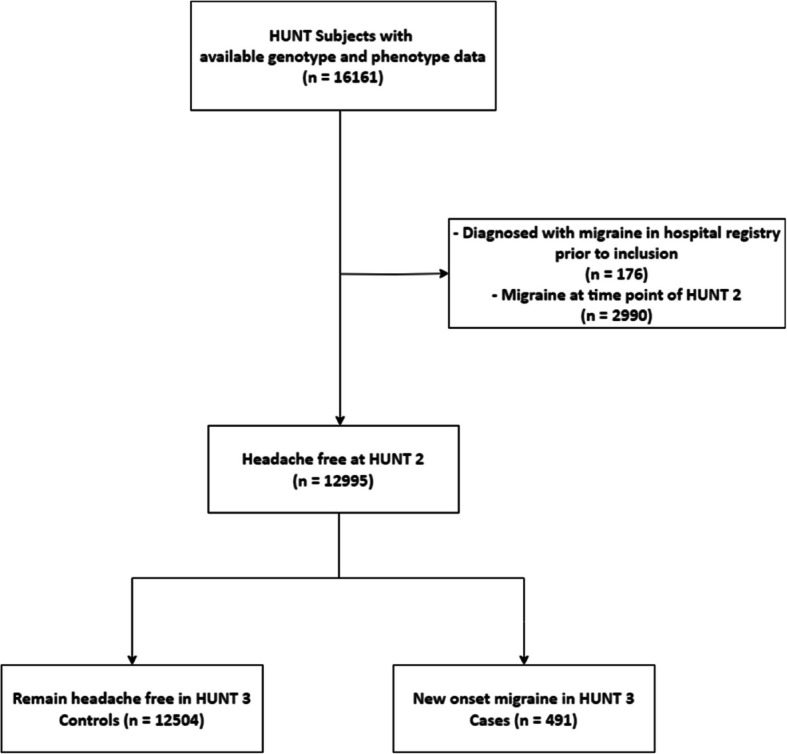
Flow-chart of patient cohort. HUNT = The Trøndelag Health Study

In total 16,161 participants had available genotypic and clinical data in HUNT2 and available migraine phenotype data in HUNT2 and HUNT3. Among these, 2,990 participants reported migraine in HUNT2 and were excluded. Further 176 were excluded based on a migraine diagnosis in the national Hospital registry. Thus, 12,995 individuals were headache-free at the time point of HUNT2 and were included in the predictive modelling. Among these, 491 (4%) reported migraine in HUNT3 and 12,504 individuals remained headache-free in HUNT3. Among those reporting migraine in HUNT3, 145 were categorized as migraine with aura. A flow-chart of the participant cohort is shown in Fig. 2. The number and percentage of individuals with new-onset migraine in the training, validation and test sets were 347 (3.8%), 106 (3.4%) and 38 (4.8%), respectively. The individuals with new onset migraine were generally younger (38 years), and more often women (63%) as compared to the controls (mean age 50 years and 48% women). The proportion of married individuals among the cases was 55% as compared to 72% among the controls. The distribution of comorbidities was relatively similar among the groups. Of note, the prevalence of musculoskeletal pain, anxiety and depression were comparable among the groups. Participant demographics are summarized in Table 1.

### Predictive performance

MICE proved to be the best imputer for variables missing at random based on its impact on the model scoring metrics. Likewise, representing the genotype data only as integers (i.e. converting imputed dosages to the nearest integers) led to the best performance. None of the resampling strategies adequately improved the imbalanced classification accuracy. Therefore, we did not resample the data and instead relied on balanced accuracy as a scoring metric for evaluating imbalanced performance.

**Fig. 3:**
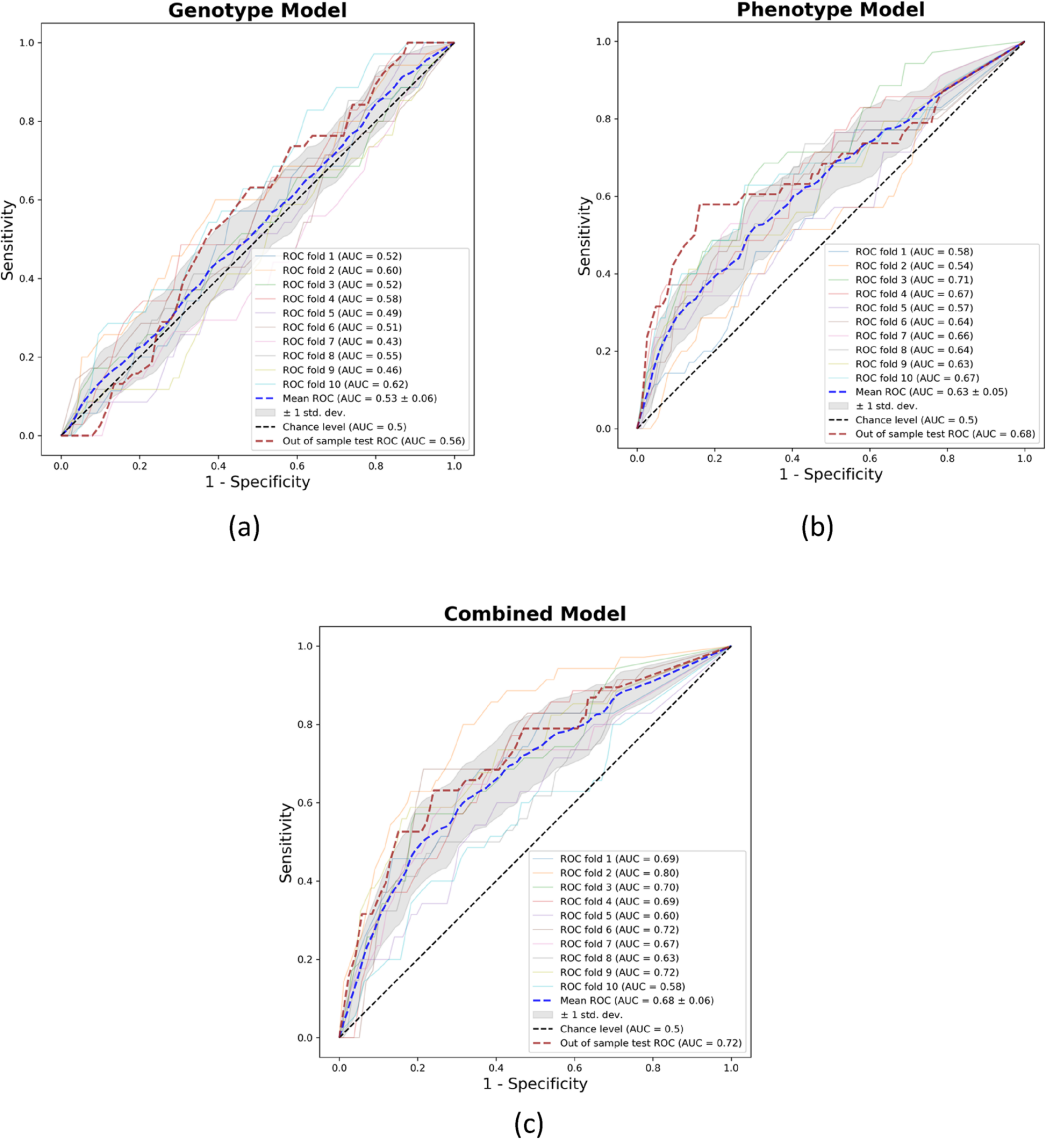
Receiver operating characteristics curves for the top performing models. The ten-fold cross validation with standard deviation (SD) is depicted as the blue line (mean) and shaded gray area (± 1SD). The dark maroon line represents the test set ROC curve. The test set performance was faithful to the training set. The dotted black line represents AUC for classification by chance given a random and equal distribution of headache data. ROC = receiver operating characteristics; AUC = area under the curve

For each of the cases, the top performing models were based on the decision tree classifier. When using only genotypic data, the top model achieved a mean cross-validated AUC of 0.53 (SD = 0.06) and a held-out test set AUC of 0.56 (95% CI 0.47 to 0.66). Using only general clinical data, the best model achieved a mean cross-validated AUC of 0.63 (SD = 0.05) and a held-out test set AUC of 0.68 (95% CI 0.58 to 0.76). The combination of genotypic and clinical data yielded the best overall performance, achieving a mean cross-validated AUC of 0.68 (SD = 0.06) and a held-out test set AUC of 0.72 (95% CI 0.63 to 0.80). Figure 3 shows ROC curves of the three models. Balanced accuracy, sensitivity, and specificity of the combined data was 0.64 (SD = 0.05), 0.63 (SD = 0.02) and 0.63 (SD = 0.02) respectively, and in the test set, it was 0.64, 0.70 and 0.71. Combining genetic and clinical features in an additive fashion using voting classifier provided a mean cross-validated AUC of 0.58 (SD = 0.03) and test set AUC of 0.67 (95% CI 0.60 to 0.74). The number and percentage of individuals with new-onset migraine in the training, validation and test sets were 347 (3.8%), 106 (3.4%) and 38 (4.8%), respectively. The AUC, balanced accuracy, sensitivity and specificity of the top performing models are reported in Table 2.

**Table 2:** Training and test set performance of the best models. For the training set the mean of each fold ± 1SD is reported

Performance Metrics	Genotype model, mean ± 1SD (test)	Phenotype model, mean ± 1SD (test)	Combined model, mean ± 1SD (test)	Additive model, mean ± 1SD (test)
AUC	0.53 ± 0.06 (0.56)	0.63 ± 0.05 (0.68)	0.68 ± 0.06 (0.72)	0.58 ± 0.03 (0.67)
Balanced accuracy	0.53 ± 0.04 (0.54)	0.59 ± 0.04 (0.65)	0.64 ± 0.05 (0.64)	0.56 ± 0.03 (0.63)
Sensitivity	0.59 ± 0.03 (0.55)	0.60 ± 0.02 (0.62)	0.63 ± 0.02 (0.70)	0.69 ± 0.01 (0.70)
Specificity	0.60 ± 0.03 (0.55)	0.60 ± 0.02 (0.62)	0.63 ± 0.02 (0.71)	0.61 ± 0.02 (0.71)

*SD* Standard deviation

### Model explainability

**Fig. 4:**
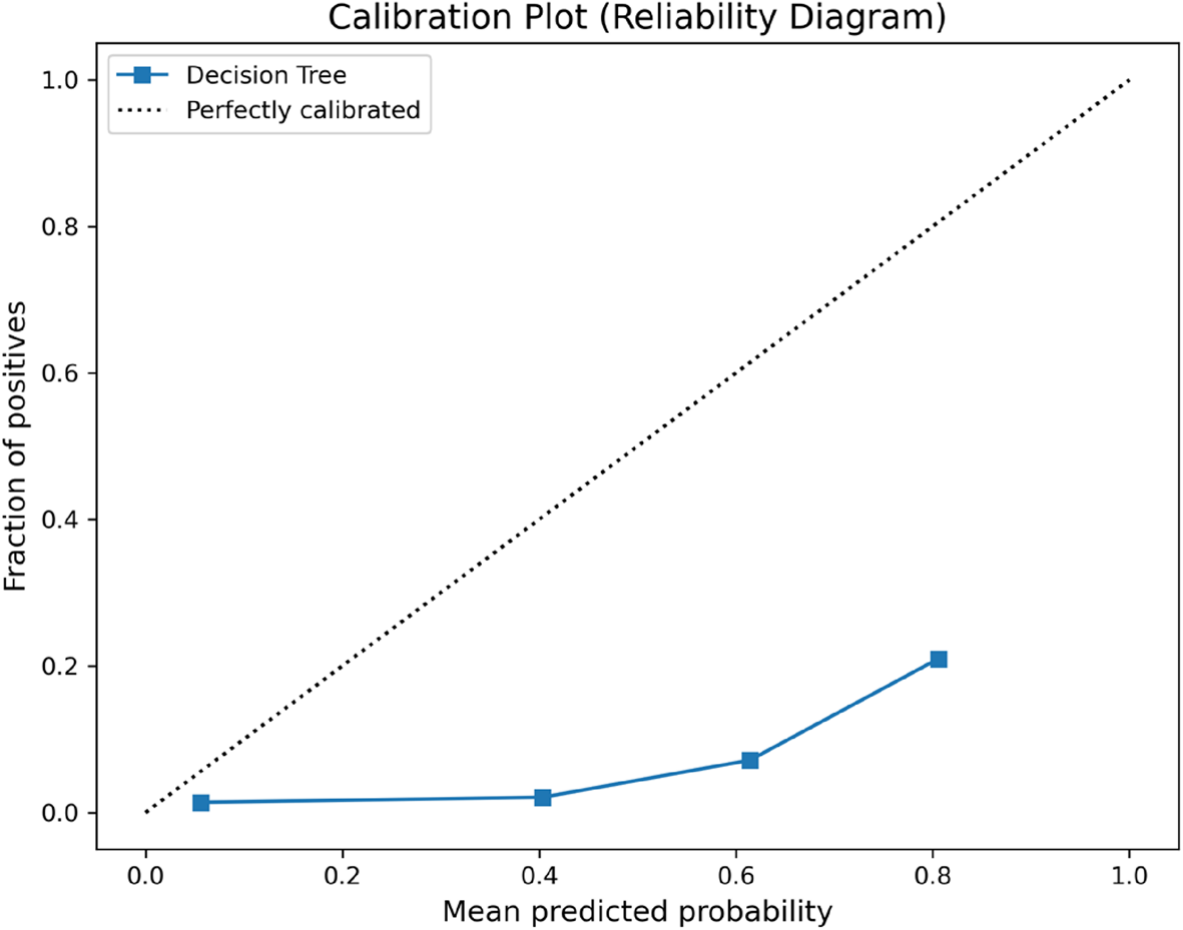
Calibration plot. Calibration plot visualizing the accuracy of the prediction of models. Here, mean predicted probabilities are plotted against the fraction of true values. The Decision Tree model’s predictions deviate significantly from the ideal diagonal line (dotted), indicating poor probability calibration—suggesting the predicted probabilities align suboptimally with the observed outcomes

**Fig. 5:**
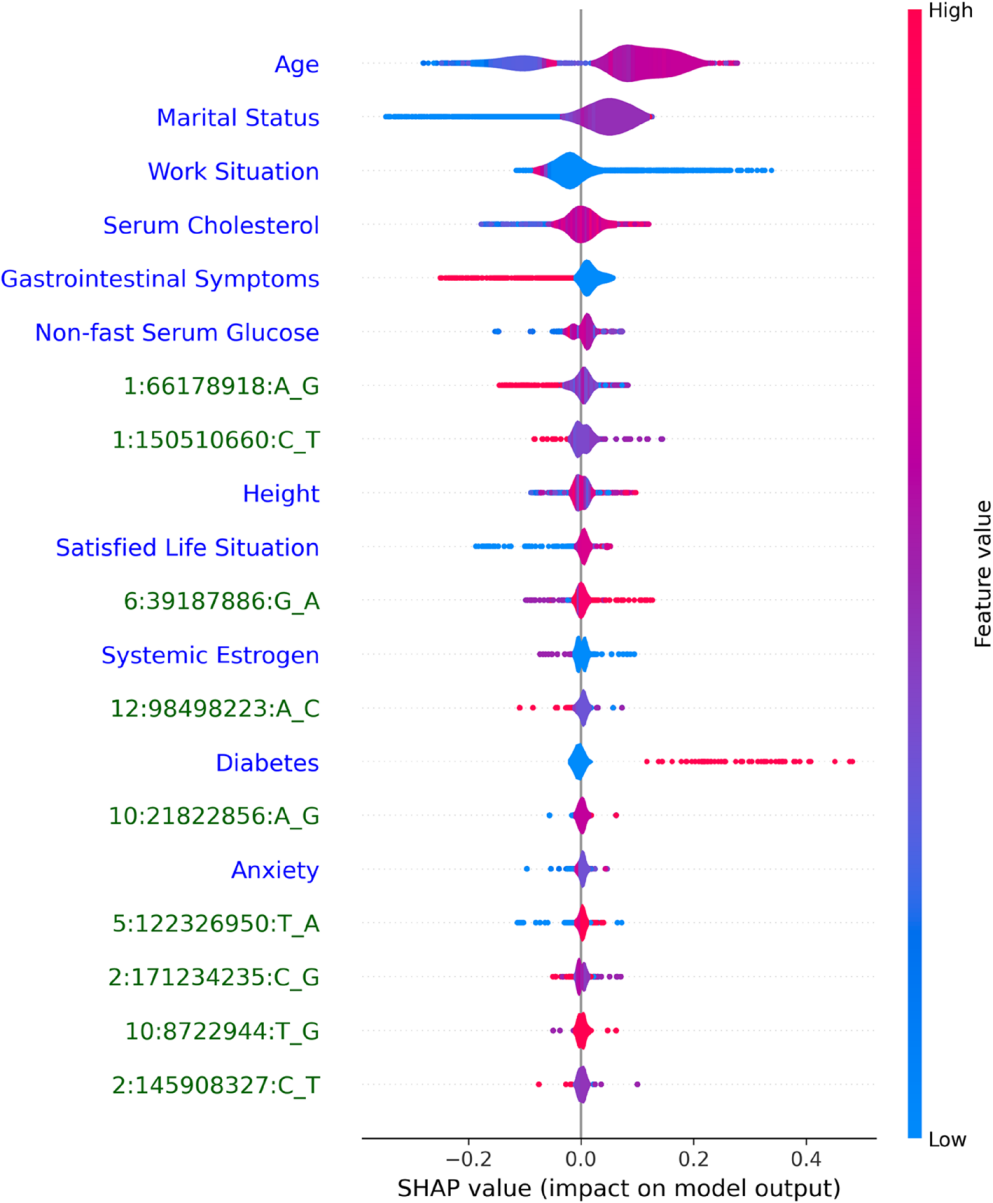
SHAP summary plot of the 20 most important features for the combined model. The notation “Chromosome: Position: ReferenceAllele_AlternateAllele” is used to state the genetic variants positions

Figure 4 is a calibration plot showing the calibration of the model against the observed values. The SHAP summary plot manifests the contributions of the most important features with a clear visualization of their directional effects on model predictions (Fig. 5). This shows the vast number of characteristics the model deems significant and the impact of a high-dimensional array of clinical and genotypic data for prediction of new-onset migraine. Among them, the most important features were age, marital status, work situation, serum cholesterol and gastrointestinal symptoms; as well as certain genetic variants (Fig. 5). Middle age, being married as opposed to not being married, and not having paid work were predictors of new-onset migraine. More gastrointestinal symptoms were also a predictor of new-onset migraine. Finally, higher levels serum cholesterol and non-fasting glucose, as well as having diabetes were predictors of new-onset migraine.

## Discussion

### Principal findings

In this population-based cohort study, we developed and validated machine learning models for predicting new-onset migraine using both clinical and genotype data from the HUNT study. Our findings demonstrate that routine clinical data can predict new-onset migraine with moderate accuracy (AUC 0.68), while genetic variants alone showed limited predictive value. The combination of both data types yielded a slight improvement (AUC of 0.72), as opposed to merely combining the predictivity of the genotype and clinical models (AUC of 0.67), suggesting that there might be relevant phenotypic-genotypic interactions that partake in the new-onset of migraine.

### Interpretation

Genotype alone seems to be a relatively poor predictor of new-onset migraine. This is not surprising as the largest-to-date GWAS meta-analysis only explained 11.2% of the migraine heritability. Still, the low predictability of the genotype in our study could have been generated by at least two factors relating to the relatively high age of the study subjects: First, a higher burden of migraine risk variants seems to lead to an earlier age of onset of migraine [26, 27], hence study subjects with these variants might already have developed migraine in HUNT 2; and second, many participants probably had a migraine onset in young adult age, prior to their HUNT2 participation.

On the other hand, general sociodemographic and clinical features proved much more predictive. This is reasonable as we know there are associations between migraine and several other diseases, such as anxiety and depression [28], gastrointestinal symptoms [29], and general lifestyle [30]. The SHAP summary plot reflects these associations, identifying more gastrointestinal symptoms, low self-reported life satisfaction, and anxiety as important predictors of new-onset migraine. Middle age and being married were also predictors of new-onset migraine, which is reasonable given the typical debut of migraine in early adulthood. Interestingly, high levels of non-fasting glucose and cholesterol, and self-reported diabetes were also important predictors of new-onset migraine. Elevated levels of cholesterol have been observed in individuals with migraine in observational studies [31], but their pathophysiological link to migraine remains unclear. The literature is also indeterminate on the strength and direction of the association between migraine and diabetes [32]. Nevertheless, the observed pattern of a wide distribution of univariate weakly associated predictors supports the usefulness of using machine learning as a predictive tool in complex diseases such as migraine.

Among the 20 most important features in our model, nine were genetic variants with an established susceptibility for migraine. For a detailed biological interpretation of these variants, we refer to the 2022 GWAS meta-analysis [9]. However, it is interesting to note that these variants are not the ones with the assumed strongest association to migraine. In fact, these nine variants ranked between the 15th and 87th most important index variants as identified in the 2022 GWAS meta-analysis [9]. A possible interpretation for this is that the model captures non-additive effects between genotype and clinical data, and thus variants with a lesser magnitude of univariate association with migraine become more important only in combination with other features.

The combination of genotype and clinical data outperforms the predictive performance of using either genotype or clinical data alone. It also outperforms the model that “additively” combines the performance of the genotype only and clinical data only models via a voting classifier. This is an important finding, because it indirectly supports the notion that genotypic-phenotypic interactions partake in the new-onset of migraine. Indeed, evidence is accumulating that epigenetic mechanisms reflecting gene-environment interactions are involved in migraine progression and transformation from episodic to chronic migraine [33]. Thus, possibly genetically predisposed but migraine-free youths that are later exposed to certain medical, lifestyle or environmental exposures could have their migraine disease predicting gene expression “turned on” by the exposure. The missing heritability, i.e. the discrepancy in the observed heritability of migraine and what is detected in GWAS, is likely, at least in part, explained by such hard-to-detect gene-environment interactions [34]. This study demonstrates that machine learning can be a valuable tool in identifying the presence of such interactions. Integration of additional data, such as real-time data sources and paraclinical data [35], could improve migraine prediction models, and our understanding of gene-environment interactions, especially when paired with transparent feature importance analyses such as SHAP [36].

A considerable obstacle with predicting the new onset of migraine in this data material was the substantial class imbalance. Among 12,995 initially headache free individuals, only 491 had a new-onset migraine in the 9-to-13-year observation period. We addressed this imbalance by evaluating several resampling techniques and appropriate scoring metrics. While SMOTE has proven valuable for imbalanced datasets [37], in this material SMOTE did not improve classification accuracy, but rather led to the classification of the synthesized data. Thus, the most important factor to counter the imbalance was the choice of appropriate scoring metrics. Still, the balanced accuracy was generally poorer than the AUC, and the sensitivity was inferior to the specificity, indicating that the models tend to predict the majority class. The incidence observed in this study of 491 individuals among 12,995 over an approximate 10-year period seems to be somewhat lower than in other populations [38]. This might be explainable by the relatively high mean age of the population.

### Strengths and limitations

The main strengths of this paper are the large population-based sample, the rich data set, the exhaustive evaluation of different machine learning approaches and the clearly defined out of sample testing. The subjects were kept strictly separated between the training and test sets which mitigates data leakage and overfitting and increases the generalizability with a robust out of sample estimate. Important limitations of this study include the class imbalance (the small number of positive outcomes those developing migraine), and the uncertainty in phenotype assignment (misclassification). This uncertainty was indeed emphasized by the fact that several individuals had received a diagnosis of migraine prior to HUNT2 but still reported that they were headache-free. Moreover, the fact that our observation window was between 9 and 13 years means that some individuals might convert to migraine in the future and thus were falsely classified as remaining headache-free. Still, previous validations of the method of phenotype assignment used showed near perfect sensitivity, and moderate specificity [18, 19]. Thus, it is unlikely that the number of undetected false positive new-onset migraine is high. Another limitation is the potential bias of including age as a feature in the model. Because migraine incidence peaks in young adulthood [38], those that were at older age during HUNT2 had likely already surpassed the age at which a migraine would debut, thus not contributing as an individual with a potential of new-onset migraine. Moreover, other features associated with age such as marital status, work situation and serum cholesterol, could further corroborate the bias. Future studies should incorporate more robust methods for case verification to ensure accurate classification and enhance the reliability of the results. Missing data is an inevitable limitation when working with large cohort data spanning multiple time-points and data sources. To counter this, we made a rigorous evaluation of each feature’s reason for missingness, and imputed accordingly with appropriate methods–of course, imputations can always introduce bias [39].

## Conclusion

The new onset of migraine in adult age is poorly predicted by genotypic data alone, however, routine demographic and clinical data (mostly non-headache-related) correctly predict the new onset of migraine in approximately 2 out of 3 cases. Integration of genotype and clinical data in the same model provides improvements in predictions, suggesting that there are genotypic-phenotypic interactions that contribute to the new- onset of migraine. Future investigations should explore different genotypic phenotypic combinations in different age groups investigate migraine subtypes and disease remission.

## Supplementary Information

Supplementary Material 1.

## Data Availability

The datasets used in this study contains personal sensitive information and is not publicly available.
