# Supplementary material for: Prediction of new-onset migraine using clinical-genotypic data from the HUNT Study: a machine learning analysis
Source: J Headache Pain. 2025 Apr 7;26(1):70. doi: 10.1186/s10194-025-02014-2 (PMC11977938; doi:10.1186/s10194-025-02014-2)
Supplement: Supplementary file 1 — Supplementary Material 1. [file 10194_2025_2014_MOESM1_ESM.docx]

**Supplementary table A.** List of all the variables

| **Category** | **Variable name** |
| --- | --- |
|  | Age  Sex |
| Clinical | Height Weight Waist Circumference Hip Circumference Body mass index Systolic blood pressure  Diastolic blood pressure Pulse Serum Cholesterol Serum HDL cholesterol Serum Triglycerides Non-fast serum glucose  Blood HbA1c |
| Socio-economic | Marital status Education |
| Work | Work Situation Work Paid Work Trade Work Home Work Education Military  Work Unemployed Work Retired Work Time Weekly Work Shift |
| Family history | Stroke 1^st^ degree relative Cardiac infarction 1^st^ degree relative Diabetes 1^st^ degree relative |
| Self-reported health | Self-reported health |
| Cardio-vascular | Myocardial infarction ever Angina ever Stroke ever Diabetes ever Tachycardia last year |
| Lung | Asthma ever Dyspnea last year |
| Thyroid | Hyperthyroid ever Hypothyroid ever |
| Neurologic | Epilepsy ever |
| Gastro-intestinal symptoms | Nausea last year Heartburn last year Diarrhea last year Constipation last year |
| Gynecological | Age at 1^st^ menstruation Still menstruation |
| Birth control | Using birth control pills Used systemic estrogens |
| Musculoskeletal | Musculoskeletal pain  Neck pain  Activity limitation pain  Fibromyalgia  Rheumatoid arthritis  Arthrosis  Spondylarthritis |
| Cancer | Cancer ever |
| Mental health | Conor mental health index  HADS anxiety sub score  HADS depression sub score  HADS total  Satisfied with life situation |
| Medications | Asthma medication  Blood pressure medication |
| Stimulants | Tobacco smoke exposure as child  Tobacco smoke exposure as adult  Tobacco smoking status  Never alcohol drinker  No. of alcohol units per month |
| Exercise | Frequency of light exercise  Frequency of hard exercise |
| Sleep | Insomnia last month  Sleeplessness in the morning last month |

HADS = Hospital Anxiety and Depression Scale

**Supplementary table B.** Imputation strategies for the missing data

| **Variable** | **Brief Assessment** | **Imputation Strategy** |
| --- | --- | --- |
| **Education** | - - 61,760 responds out of 65,228 responders to the questionnaire (94.7%).   - More probable that people with low education did not answer than people with higher education.   - Most missingness is missingness not at random (MNAR) – low vs high education. | - - Put non-responders in category with lowest education. |
| **Work situation** | - - Hard to calculate number and percentage of responders/non-responders as participants could choose multiple answers.   - More probable that unemployed people did not answer than people with employment => missingness not at random (MNAR).   - Make variable binary (paid vs unpaid work). | - - Put non-responders in unpaid work category. |
| **Weekly work time** | - - 43,641 responds out of 65,228 responders of the questionnaire.   - Non-responders do not have paid work (they might interpret the question to only apply to people with paid work).   - Non-responders do not have a fixed work time, so they might not know how many hours they work.   - MNAR (more probable that non-responders do not have paid work). | - - Make variable binary (paid vs unpaid) and put non-responders in unpaid category. |
| **Work shift** | - - 44,186 responds out of 65,228 responders of the questionnaire.   - 21.36% of responders responded yes.   - Non-responders are more probable to not be employed (retired/student/social welfare).   - MNAR (non-responders more likely to not be employed). | - - Put non-responders in “no” category. |
| **Tachycardia last year** | - - Three options: not troubled – a little troubled – very troubled   - 57,486 responders out of the 65,228 responders to the questionnaire.   - Similar number of responders to the other (nausea, heartburn, constipation…).   - There is more probability that you do not respond if you do not have the symptom. MNAR. | - - Non-responders set to “not troubled”. |
| **Dyspnea last year** | - - 56,290 responders out of 65,228.   - Similar response rate as Tachycardia last year which is part of the same question. MNAR. | - - Non-responders set to “not troubled”. |
| **Never alcohol drinker** | - - Non-responders are probably drinking alcohol.   - MNAR. | - - Set non-responders as ‘Drinking ones’ |
| **Still menstruation** | - - Females 20-69 were asked and females > 70 were asked how old they were when they stopped menstruating.   - If they answered this, they were set to not still menstruating.   - 28,485 responders out of 55,452 total responders of which 30,314 were female. Some questionnaire 2 respondents didn’t participate in questionnaire 1.   - Check if there is any missingness due to gender (men not classified as -1).   - Is any missingness due to age > 70? | - - Put people under mean menopause age as 1 (still menstruation) and over as 0 (not anymore). |
| **Systemic estrogens** | - - 25,732 responders out of 55,452 total responders of which 30,314 were female   - Check if there is any missingness due to gender (men not classified as -1) and any due to age < 30   - In age group < 30 a total of 6,522 subjects. Of these, 1,423 are missing it.   - A total of 0.1% have answered Yes. Of the missing it is likely that females should be 0 and men should be -1   - After imputing this, we still have missingness of 3,131. | - - MNAR. Put non-responders in “never” group. |
| **Musculoskeletal neck pain** | - - Musculoskeletal pain in neck (MSPaNec) is conditioned on musculoskeletal pain last year (MSPaLY).   - If MSPaLY is answered no MSPaNec is skipped in the questionnaire and are categorized as “no” in MSPaNec.   - If MSPaLY is answered yes, the subjects need to answer either yes or no on MSPaNec.   - HUNT2Q1. If pain/and or stiffness in your muscles and limbs, where did you have these health problems?   - Very good response rate to MSPaLY! The missingness is probably due to strategy in categorizing.   - After discussion with medical and AI experts, it seems highly likely that most missingness is due to people answering yes to the conditioned question (MSPaLY) and not crossing off for either Yes/No in MSPaNec. | - - MNAR => MI cannot be done.   - Put all non-responders in category “no”. |
| **Satisfied with life situation** | - - 63,745 out of 65,228 responded.   - MNAR (For non-responders, this might be a “touchy” subject for people who are dissatisfied so the ones who didn’t respond might be more dissatisfied than the ones who responded). | - - Set non-responders as dissatisfied. |
| **Frequency of hard exercise** | - - Non-responders are probably in little physical activity.   - MNAR. | - - Set non-responders as 0. |

All the variables above are still 10% missing except a few. Now, the strategy for imputing the variables with missingness (<10%) are shown below.

| **Variable** | **Brief Assessment** | **Imputation Strategy** |
| --- | --- | --- |
| **Height**  **Weight**  **Waist circumference**  **Hip circumference**  **Body mass index**  **Pulse**  **Marital status** | - - Very few missing values.   - Missing at random (MAR). | - - Multiple imputation. |
| **Systolic blood pressure**  **Diastolic blood pressure** | - - Blood pressure is probably higher in the group of missingness.   - Very few missing values. So probably MAR. | - - Multiple imputation. |
| **Serum cholesterol**  **Serum HDL cholesterol**  **Serum Triglycerides**  **Non-fast serum glucose** | - - Very few missing values.   - MAR. | - - Multiple imputation. |
| **Current health status**  **Mental Health index** | - - Very few missing values.   - The ones who didn’t respond might have bad heath/mental condition than the ones who responded. MNAR. | - - Impute non-responders to 0. |
| **Myocardial infraction** | - - Non responders might not have the heart attack. But very few missing value.   - Probably missing at random. | - - Multiple imputation. |
| **Apoplexy**  **Diabetes**  **Angina pectoris**  **Epilepsy**  **Cancer**  **Asthma** | - - Non responders might not have these diseases.   - MNAR. | - - Impute by ‘No’. |
| **Family history**  **Apoplexy** **Cardiac infarction** **Diabetes** | - - Non responders might not have these diseases in 1^st^ degree relative. MNAR. | - - Impute missingness with ‘None’. |
| **Hyperthyroid**  **Hypothyroid** | - - High and low value of Thyroid hormones. Non responders might not have it. MNAR. | - - Categorize in ‘Not have’ zone. |
| **Gastrointestinal symptoms**  **Nausea**  **Heartburn**  **Diarrhea**  **Constipation** | - - Very few missing values. Non responders might not have it. MNAR. | - - Impute non responders to 0. |
| **Age at 1^st^ menstruation** | - - Maybe did not remember. Maybe never had menstruation. MNAR. | - - Put non-responders in “never” group. |
| **Birth control pills** | - - Non responders might not have it. MNAR. | - - Impute non responders to 0. |
| **Musculoskeletal pain last year**  **Musculoskeletal neck pain**  **Activity limitation pain** | - - Non responders might not have it. MNAR. | - - Impute non responders to 0. |
| **Fibromyalgia**  **Rheumatoid arthritis**  **Arthrosis**  **Spondylarthritis** | - - Non responders are more likely to not have these diseases. MNAR. | - - Set missingness/non responders to 0. |
| **HADS anxiety sub score**  **HADS depression sub score**  **HADS total** | - - Very few missing values. MAR. | - - Multiple imputation. |
| **Asthma medication**  **Blood pressure medication** | - - Non responders might not have it. MNAR | - - MNAR. Non responders to 0. |
| **Tobacco smoke exposure as child**  **Tobacco smoking status** | - - Very few missing values. MAR. | - - Multiple imputation. |
| **Insomnia last month**  **Sleeplessness in the morning last month** | - - Non responders might not have it. MNAR | - - Set missingness to 0. |

HDL = High-density lipoproteins

HADS = Hospital Anxiety and Depression Scale

**International Headache Genetics Consortium**

Verneri Anttila^1,2,3^, Ville Artto^4^, Andrea C Belin^5^, Anna Bjornsdottir^6^, Gyda Bjornsdottir^7^, Dorret I Boomsma^8^, Sigrid Børte^9,10,11^, Mona A Chalmer^12^, Daniel I Chasman^13,14^, Bru Cormand^15^, Ester Cuenca-Leon^16^, George Davey-Smith^17^, Irene de Boer^18^, Martin Dichgans^19,20^, Tonu Esko^21^, Tobias Freilinger^22,23^, Padhraig Gormley^24^, Lyn R Griffiths^25^, Eija Hämäläinen^26^, Thomas F Hansen^12,27^, Aster VE Harder^18,28^, Heidi Hautakangas^26^, Marjo Hiekkala^29^, Maria G Hrafnsdottir^30^, M. Arfan Ikram^31^, Marjo-Riitta Järvelin^32,33,34,35^, Risto Kajanne^26^, Mikko Kallela^4^, Jaakko Kaprio^26^, Mari Kaunisto^29^, Lisette JA Kogelman^12^, Espen S Kristoffersen^36,37,38^, Christian Kubisch^39^, Mitja Kurki^40^, Tobias Kurth^41^, Lenore Launer^42^, Terho Lehtimäki^43^, Davor Lessel^39^, Lannie Ligthart^8^, Sigurdur H Magnusson^7^, Rainer Malik^19^, Bertram Müller-Myhsok^44^, Carrie Northover^45^, Dale R Nyholt^46^, Jes Olesen^12^, Aarno Palotie^26,47^, Priit Palta^26^, Linda M Pedersen^48^, Nancy Pedersen^49^, Matti Pirinen^26,50,51^, Danielle Posthuma^52^, Patricia Pozo-Rosich^53^, Alice Pressman^54^, Olli Raitakari^55,56,57^, Caroline Ran^5^, Gudrun R Sigurdardottir^6^, Hreinn Stefansson^7^, Kari Stefansson^7^, Olafur A Sveinsson^30^, Gisela M Terwindt^18^, Thorgeir E Thorgeirsson^7^, Arn MJM van den Maagdenberg^18,28^, Cornelia van Duijn^58^, Maija Wessman^29,26^, Bendik S Winsvold^48,9,59^, John-Anker Zwart^48,9,10^

^1^Analytical and Translational Genetics Unit, Department of Medicine, Massachusetts General Hospital and Harvard Medical School, Boston, Massachusetts, USA, ^2^Program in Medical and Population Genetics, Broad Institute of MIT and Harvard, Cambridge, Massachusetts, USA, ^3^Stanley Center for Psychiatric Research, Broad Institute of MIT and Harvard, Cambridge, Massachusetts, USA, ^4^Department of Neurology, Helsinki University Central Hospital, Helsinki, Finland, ^5^Department of Neuroscience, Karolinska Institutet, Stockholm, Sweden, ^6^Neurology private practice, Laeknasetrid, Reykjavik, Iceland, ^7^deCODE genetics/Amgen Inc., Reykjavik, Iceland, ^8^Netherlands Twin Register, Department of Biological Psychology, Vrije Universiteit, Amsterdam, the Netherlands, ^9^K.G. Jebsen Center for Genetic Epidemiology, Department of Public Health and Nursing, Faculty of Medicine and Health Sciences, Norwegian University of Science and Technology, Trondheim, Norway, ^10^Institute of Clinical Medicine, Faculty of Medicine, University of Oslo, Oslo, Norway, ^11^Research and Communication Unit for Musculoskeletal Health, Department of Research, Innovation and Education, Division of Clinical Neuroscience, Oslo University Hospital, Oslo, Norway, ^12^Danish Headache Center, Department of Neurology, Copenhagen University Hospital, Copenhagen, Denmark, ^13^Department of Medicine, Division of Preventive Medicine, Brigham and Women's Hospital, Boston, Massachusetts, USA, ^14^Harvard Medical School, Boston, Massachusetts, USA, ^15^Department of Genetics, Spain Centre for Biomedical Network Research on Rare Diseases, University of Barcelona, Barcelona, Spain, ^16^Pediatric Neurology Research Group, Vall d'Hebron Research Institute, Barcelona, Spain, ^17^University of Bristol/Medical Research Council Integrative Epidemiology Unit, University of Bristol, Bristol, UK, ^18^Department of Neurology, Leiden University Medical Centre, Leiden, the Netherlands, ^19^Institute for Stroke and Dementia Research, University Hospital, LMU Munich, Munich, Germany, ^20^Munich Cluster for Systems Neurology, Munich, Germany, ^21^Estonian Biobank Registry, the Estonian Genome Center, University of Tartu, Tartu, Estonia, ^22^Department of Neurology, Klinikum Passau, Passau, Germany, ^23^Department of Neurology and Epileptology, Hertie Institute for Clinical Brain Research, University of Tuebingen, Tuebingen, Germany, ^24^GSK Inc., Cambridge, Massachusetts, USA, ^25^Centre for Genomics and Personalised Health, Queensland University of Technology, Brisbane, Queensland, Australia, ^26^Institute for Molecular Medicine Finland, Helsinki Institute of Life Science, University of Helsinki, Helsinki, Finland, ^27^Novo Nordic Foundation Center for Protein Research, Copenhagen University, Copenhagen, Denmark, ^28^Department of Human Genetics, Leiden University Medical Centre, Leiden, the Netherlands, ^29^Folkhälsan Research Center, Helsinki, Finland, ^30^Landspitali University Hospital, Reykjavik, Iceland, ^31^Department of Epidemiology, Erasmus University Medical Center, Rotterdam, the Netherlands, ^32^Department of Epidemiology and Biostatistics, MRC-PHE Centre for Environment and Health, School of Public Health, Imperial College London, London, UK, ^33^Center for Life Course Health Research, Faculty of Medicine, University of Oulu, Oulu, Finland, ^34^Unit of Primary Health Care, Oulu University Hospital, OYS, Oulu, Finland, ^35^Department of Life Sciences, College of Health and Life Sciences, Brunel University London, London, UK, ^36^Research and Communication Unit for Musculoskeletal Health, Department of Research, Innovation and Education, Division of Clinical Neuroscience, Akershus University Hospital and University of Oslo, Oslo, Norway, ^37^Department of General Practice, Institute of Health and Society, University of Oslo, Oslo, Norway, ^38^Department of Neurology, Akershus University Hospital, Lørenskog, Norway, ^39^Institute of Human Genetics, University Medical Center Hamburg-Eppendorf, Hamburg, Germany, ^40^Psychiatric and Neurodevelopmental Genetics Unit, Department of Medicine, Massachusetts General Hospital, Boston, Massachusetts, USA, ^41^Institute of Public Health, Charité – Universitätsmedizin, Berlin, ^42^Laboratory of Epidemiology and Population Sciences, Intramural Research Program, National Institute on Aging, Bethesda, Maryland, USA, ^43^Department of Clinical Chemistry, Fimlab Laboratories, and Finnish Cardiovascular Research Center - Tampere, Faculty of Medicine and Health Technology, Tampere University, Tampere, Finland, ^44^Max Planck Institute of Psychiatry, Munich, Germany, ^45^23&Me Inc., Mountain View, California, USA, ^46^School of Biomedical Sciences, Faculty of Health, Centre for Genomics and Personalised Health, Centre for Data Science, Queensland University of Technology, Brisbane, Queensland, Australia, ^47^University of Helsinki, Helsinki, Finland, ^48^Department of Research, Innovation and Education, Division of Clinical Neuroscience, Oslo University Hospital, Oslo, Norway, ^49^Department of Medical Epidemiology and Biostatistics, Karolinska Institutet, Stockholm, Sweden, ^50^Department of Mathematics and Statistics, University of Helsinki, Helsinki, Finland, ^51^Department of Public Health, University of Helsinki, Helsinki, Finland, ^52^Department of Complex Trait Genetics, Center for Neurogenomics and Cognitive Research, Neuroscience Campus Amsterdam, VU University, Amsterdam, The Netherlands, ^53^Headache Unit, Neurology Department, Vall d'Hebron University Hospital, Barcelona, Spain, ^54^Sutter Health, Sacramento, California, USA, ^55^Centre for Population Health Research, University of Turku, Turku University Hospital, Turku, Finland, ^56^Research Centre of Applied and Preventive Cardiovascular Medicine, University of Turku, Turku, Finland, ^57^Department of Clinical Physiology and Nuclear Medicine, Turku University Hospital, Turku, Finland, ^58^Department of Epidemiology, Erasmus University Medical Centre, Rotterdam, the Netherlands, ^59^Department of Neurology, Oslo University Hospital, Oslo, Norway
